# Supplementary material for: Mezcal Characterization Through Sensory and Volatile Analyses
Source: Foods. 2025 Jan 26;14(3):402. doi: 10.3390/foods14030402 (PMC11816729; doi:10.3390/foods14030402)
Supplement: Supplementary file 1 [file foods-14-00402-s001.zip › foods-3369117-supplementary.pdf]

FREE CHOICE PROFILING FORMAT

Name Date

Now you will taste a series of samples, please score your elicited attribute from 0 to 10 according to the intensity that you perceive. Thank you

Sample: 345

Flavor attribute	Score
Odor attribute	Score

Name **Date**

Evaluation of Mezcal Samples

Please indicate the perceived intensity of each one of the odor and flavor descriptors.

**Odor**	**399**	**316**	**344**	**327**	**600**	**512**	**613**	**428**	**387**	**560**
Alcohol										
Citric										
Earthy										
Herbal										
Floral										
Fruity										
Menthol herbs										
Smoked										
Spices										
Wood										

**Flavor**	**399**	**316**	**344**	**327**	**600**	**512**	**613**	**428**	**387**	**560**
Alcohol										
Astringent										
Bitter										
Citric										
Earthy										
Floral										
Fresh										
Fruity										
Maguey										
Mineral salts										
Smoked										
Spices										
Wood										
